# FF3D: A Rapid and Accurate 3D Fruit Detector for Robotic Harvesting

**DOI:** 10.3390/s24123858

**Published:** 2024-06-14

**Authors:** Tianhao Liu, Xing Wang, Kewei Hu, Hugh Zhou, Hanwen Kang, Chao Chen

**Affiliations:** 1 Faculty of Engineering, Monash University, Clayton, VIC 3800, Australia; tianhao.liu@monash.edu (T.L.); hanwen.kang@outlook.com (H.K.); 2 CSIRO’s Data61, Level 5/13 Garden St, Eveleigh, NSW 2015, Australia; 3 College of Engineering, South China Agriculture University, Guangzhou 510070, China

**Keywords:** deep learning, 3D vision, smart agriculture, robotic harvesting

## Abstract

This study presents the Fast Fruit 3D Detector (FF3D), a novel framework that contains a 3D neural network for fruit detection and an anisotropic Gaussian-based next-best view estimator. The proposed one-stage 3D detector, which utilizes an end-to-end 3D detection network, shows superior accuracy and robustness compared to traditional 2D methods. The core of the FF3D is a 3D object detection network based on a 3D convolutional neural network (3D CNN) followed by an anisotropic Gaussian-based next-best view estimation module. The innovative architecture combines point cloud feature extraction and object detection tasks, achieving accurate real-time fruit localization. The model is trained on a large-scale 3D fruit dataset and contains data collected from an apple orchard. Additionally, the proposed next-best view estimator improves accuracy and lowers the collision risk for grasping. Thorough assessments on the test set and in a simulated environment validate the efficacy of our FF3D. The experimental results show an AP of 76.3%, an AR of 92.3%, and an average Euclidean distance error of less than 6.2 mm, highlighting the framework’s potential to overcome challenges in orchard environments.

## 1. Introduction

Instance-aware perception is an important task in modern smart agriculture. In recent years, considerable efforts have been dedicated to achieving fully autonomous harvesting by determining the precise position of fruits. The emergence of deep learning has led to significant advancements and breakthroughs across various domains. Specifically, in computer vision, deep learning-based methods have demonstrated remarkable performance in tasks ranging from image classification and object detection to image generation [1,2,3,4]. These achievements have greatly accelerated the development of fruit detection. A number of works have achieved good accuracy in fruit detection and segmentation tasks [5,6,7]. Despite the significant progress made in terms of 2D performance by these studies, it does not directly reflect their perceptual capabilities in robot harvesting tasks. This is because robot harvesting requires accurate positioning of the target objects, and when occlusion levels are high, image-based methods cannot provide precise 3D positional information. High occlusion is a particularly common issue that robots encounter in autonomous harvesting tasks [8]. Therefore, research aimed at addressing the challenge of 3D detection of small objects under occlusion in the field of agricultural robotics is valuable.

Significant advancements in 3D detection technology have been made in other fields, such as the autonomous driving domain, particularly in detecting and tracking big obstacles, and these technologies perform exceptionally well in open-road environments. However, in the field of agricultural robotics, especially in fruit detection tasks, the challenges are unique [9]. Most fruit harvesting robots require accurate target position for robot manipulation [10,11,12,13,14,15]. Agricultural environments often feature vegetation, trees, branches of fruit trees, and other complex structures and factors that may result in small fruits being obscured, making their precise detection in 3D space exceptionally challenging. Therefore, it is crucial to specifically address the research challenges associated with fruit detection by agricultural robots under occlusion. From a theoretical perspective, this involves enhancing the perceptual capabilities of existing algorithms for multi-modal information in occluded environments and improving their ability to estimate occlusion situations.

This study introduces an innovative, fully convolutional 3D fruit detector that directly utilizes colorized point clouds as an input. Meanwhile, we introduce a next-best view (NBV) algorithm based on anisotropic Gaussians to optimize the process of multiple scans. This approach uniquely integrates both graphical and geometrical information, leveraging the benefits of detailed point-cloud geometry and realistic color attributes from RGB images. We conducted an in-depth analysis of state-of-the-art 3D detection networks. Based on the detailed analysis of relevant 3D detection networks, we constructed a 3D detection network based on FCAF3D [16] by leveraging the foundational structure of FCAF3D and incorporating our newly introduced occlusion-aware loss function; our model exhibits superior performance compared to state-of-the-art models. This is particularly evident in small object detection within highly occluded environments. It is noteworthy that, due to the limited information obtained from a single viewpoint in complex occlusion environments, we emphasized the importance of multiple scans in our research. In the process of multiple scans, we efficiently and accurately identify fruits in the scene with fewer detection instances by utilizing a novel obstacle representation based on anisotropic Gaussian. This approach draws inspiration from 3D Gaussian splatting [17], contrasting with the conventional method of using discrete point clouds or voxels to represent obstacles in the scene. Leveraging this obstacle representation, we can quickly find the next-best view (NBV). The specific contributions of this work include:

- Proposition and development of a new occlusion-aware 3D fruit detection network.
- Proposition and development of a new anisotropic Gaussian-based next-best view estimation for multi-view detection.
- Demonstration and quantitative analysis of different 3D detection methods in a manually labeled dataset and in a simulated environment.

This article will follow the sequential order of introducing related works, problem definition, methodology, experiments and results, and conclusion. In the methodology section, we will provide a detailed exposition in the order of 3D detection neural network, multi-view detection, and implementation specifics pertaining to this study. In the experiments and results section, a comparative analysis of the apple localization performance is presented, both in the test set and simulated scenarios.

## 2. Related Works

Many fragile fruits and vegetables, such as apples, oranges, kiwis, bell peppers, and tomatoes, require precise handling during the harvesting process to ensure a high success rate [18,19]. Consequently, there has been a significant focus in recent years on research aimed at obtaining accurate positions of these fruits [20]. Most of these studies primarily concentrate on 2D image-based detection methods [5,6,21,22,23,24]. For instance, Reference [25] improves existing 2D detection network structures to enhance 2D detection performance, while Reference [5] estimates the actual positions of fruits by combining 2D fruit locations with depth values provided by depth cameras. These studies typically operate in ideal laboratory conditions or farm environments with minimal occlusion.

However, in real orchards, occlusion is often severe, leading to a significant drop in accuracy when applying the aforementioned methods in practical applications. As a result, some studies seek to improve detection performance under complex occlusion conditions by analyzing depth information [26]. For example, Reference [23] applies F-PointNet [27] to identify clusters of fruit points and uses RANSAC fitting to determine the center of fruits. Nevertheless, existing works in this area face limitations in utilizing multiple sensor information, prominent issues being the independent processing of color images and depth information and a lack of effective fusion mechanisms. To effectively integrate color images and depth information, it is necessary to use joint processing and fusion techniques.

On the other hand, recognizing the limited information obtained from a single angle, some works aim to enhance overall precision and recall through multi-angle detection [28,29]. To enhance the efficiency of multi-view detection, some studies use NBV estimation to maximize the observable content in the next perspective [29]. NBV is typically measured using metrics such as information gain, reduction in uncertainty, and task completion, among others. However, due to the unordered nature of point clouds, these works commonly voxelate point clouds, using voxels to represent the detection region. Consequently, the representation of complex occlusion conditions is insufficient, as the information density drastically decreases during the voxelization process. For instance, under severe occlusion where the target crops are surrounded, observing voxels may not reveal perspectives with lighter occlusion. Therefore, NBV detection under conditions of substantial occlusion remains an unresolved challenge.

In other domains, there is also a demand for obtaining precise 3D object positions. For example, in autonomous driving tasks, acquiring accurate positions of other traffic participants is essential [30]. However, in the context of normal road scenarios, objects in the scene are typically on a flat plane. In relevant works on autonomous driving, the height information from sensors is compressed to varying degrees. This can be achieved by organizing point clouds into a bird’s-eye view plane via voxels [31] or columnar structures [32]. Such methods significantly reduce data volume while providing good position estimation on the flat plane. Nevertheless, due to the compression of height information, these methods struggle to provide accurate position estimates in scenarios where object heights are not uniform. Meanwhile, some works specifically focus on object recognition in scenes with non-uniform object heights, such as indoor object detection [16,33,34]. These methods choose to directly process raw 3D inputs or organize point clouds into 3D voxels for object detection. Among them, Reference [16] is the first fully convolutional, anchor-free method. It eliminates the need for predefined anchor points or assumptions about the shape and size of objects. It has achieved state-of-the-art results on indoor 3D object detection datasets.

## 3. Problem Statement

Our research is focused on 3D fruit detection with NBV under complex occlusion conditions, with the primary challenges lying in the 3D detection of small objects under high occlusion and accurately modeling obstacle representations under high occlusion conditions to identify NBV precisely. Existing methods currently have limitations in addressing this problem, particularly in the context of 3D fruit detection networks. Despite some progress in prior research on fruit detection, there still exists an issue of low precision and recall under high occlusion conditions. Therefore, we propose utilizing a 3D detection network based on colored point clouds combined with next-best view (NBV) strategies using anisotropic Gaussian obstacle representations.

Our approach involves a robot performing a series of scans at different views ($v_{1},v_{2},v_{3},...,v_{n}$). At each view $V_{i}$, the newly obtained fruit positions and surrounding 3D obstacle Gaussian information (refer to Section 4.3.1) are merged into the global map. Global 3D non-maximum suppression (NMS) is then applied to determine the most accurate fruit position estimation based on confidence scores. This results in multiple scanned fruits f∈F. For each scan, a next-best view $V^{*}$ needs to be determined in order to achieve the highest information gain (IG) for the next scan. In practical harvesting scenarios, finding the absolute optimal angle is often unnecessary. Instead, the goal is to quickly identify a reasonably optimal viewing angle within a given range that the robotic arm can reach. Therefore, we define IG in order to rapidly identify the next less-occluded viewpoint, prioritizing efficiency over a complete scan of the entire scene.

## 4. Materials and Methods

### 4.1. System Framework

**Figure 1:**
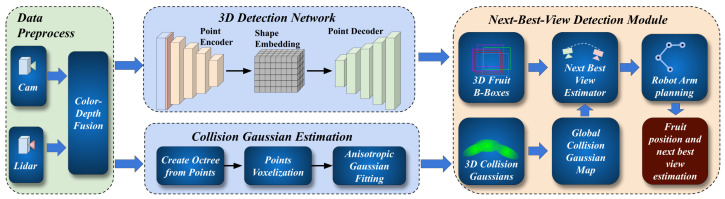
Overview of the FF3D NBV 3D fruit detection system. The 3D detection network and collision Gaussian estimation module generate fruit estimations and collision Gaussians, respectively. The NBV estimator takes those two as inputs and estimates the best view for the next scan.

The schematic representation of our FF3D is illustrated in Figure 1. The system comprises four integral modules: data preprocessing, 3D detection network, collision Gaussian estimation, and NBV estimation.

### 4.2. Network Architecture

**Figure 2:**
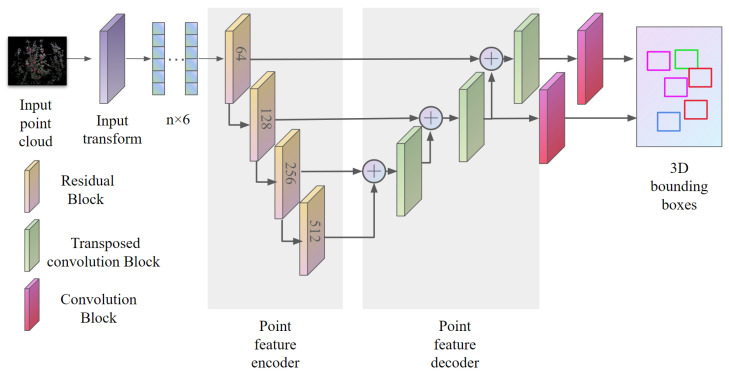
3D detector architecture.

Our network architecture is built upon FCAF3D [16]. The FCAF3D is the first fully convolutional, anchor-free 3D object detection network. It takes a set of RGB-colored points as an input and produces a set of 3D object bounding boxes as its output. Furthermore, an anchor-free head with a simple multi-level location assignment is introduced. Those features make it suitable for the detection of small objects, such as fruit. Inherited from FCAF3D, our model is composed of an encoder–decoder structure. The architecture of FF3D detection network is shown in Figure 2.

#### 4.2.1. Encoder

The encoder processes the input data and extracts high-level features. The encoder used is MinkResNet [35], with 64, 128, 256, and 512 channels in each layer. MinkResNet is a sparse, high-dimensional version of ResNet [2]. It can be considered as an adaptation of ResNet used to handle sparse inputs efficiently. In MinkResNet, a sparse tensor is used to store the input point data, as most of the space is empty. Subsequently, a generalized sparse convolution is used for sparse tensor. This encompasses both dense and sparse convolutions on the input sparse tensor.

#### 4.2.2. Decoder

The decoder is a simplified version of the GSDN decoder [36]. Due to the nature of point cloud data, the sampling point is located on the surface of the object, while the center of the object may be far from the surface. This poses a difficulty for anchor prediction. In the GSDN decoder, a transposed convolution extends a sparse point, creating an expansively dense region that can grow to arbitrary sizes, leading to the possibility of overlapping regions. The expansion from transposed convolution not only upsamples the data but also creates many empty voxels that do not contain any bounding box anchors. Pruning removes voxels with low anchor probability *P* to reduce memory usage. The probability *P* is obtained utilizing function $P_{s}\left(T,W\right)$, which takes input tensor T and 3D convolution kernel weights *W* as input.

To detect objects of different sizes, the decoder process features on each level with one sparse transposed 3D convolution and one sparse 3D convolution. The features of the higher level are passed to the lower level. In FCAF3D, each layer of the decoder is connected to a head. However, it was observed that the higher-level head does not significantly contribute to the detection of small objects. Thus, to improve the inference speed, we dropped out the heads of the third and fourth layers.

#### 4.2.3. Detection Head

The anchor-free FCAF3D head is comprised of three sparse convolutional layers that operate in parallel, and these layers share weights across different feature levels [16]. The head outputs classification probabilities, centerness, and box regression for each position (x, y, z), respectively.

Based on the head structure of FCAF3D, additional occlusion loss terms have been added, resulting in a total of four terms. The loss function of FF3D is defined as follows:

(1) $$\begin{array}{c} L=\frac{1}{n_{pos}}(L_{cls}\left(\hat{p},p\right)+L_{reg}\left(\hat{b},b\right)+L_{cntr}\left(\hat{c},c\right)\\ +L_{occ}\left(\hat{O},O\right)) \end{array}$$

where the classification loss $L_{cls}\left(\hat{p},p\right)$ is a focal loss, bounding box b(x,y,z,l,h,w); regression loss $L_{reg}\left(\hat{b},b\right)$ is (distance–intersection over union) DIoU loss; and the centerness loss $L_{cntr}\left(\hat{c},c\right)$ is binary cross-entropy loss. $\hat{p}$ and $\hat{b}$ denote the ground truth label and box respectively. The $n_{pos}$ is the number of positive samples. DIoU improves alignment by incorporating center distance, providing better gradients for faster convergence. Centerness loss prioritizes predictions close to the object’s center, enhancing model focus on accurate detections and improving training efficiency. This combined effect of DIoU and centerness leads to quicker and more precise learning. The DIoU loss term is defined as:

(2) $$L_{reg}=1-IoU+\frac{\rho^{2}\left(b,\hat{b}\right)}{c^{2}}$$

The $\rho (b,\hat{b})$ is the Euclidean distance length between the center of the *b* and $\hat{b}$, and *c* is the diagonal length of the smallest enclosing box that encompasses the two boxes [37].

The occlusion loss $L_{occ}$ is defined as follows:

(3) $$L_{occ}=\frac{1}{n}\sum_{n}|O_{i}-\hat{O_{i}}|$$

The occlusion ratio is defined as the proportion of the fruit area that is obscured or hidden by other point cloud elements in the image plane. To obtain this, we first voxelized the point cloud within the region of interest (ROI) and extracted foreground voxels based on the distance to the camera. The associated pixels on the RGB image of foreground voxels $Area_{fore_{i}}$ and fruit $Area_{Fruit_{i}}$ can be obtained using the same method described in Section 5.1. Thus, the ground truth occlusion ratio $\hat{O_{i}}$ of fruit *i* is calculated as:

(4) $$\hat{O_{i}}=\frac{Area_{Fruit_{i}}\cap Area_{fore_{i}}}{Area_{Fruit_{i}}}$$

The additional occlusion loss term serves to educate the model in handling various degrees of occlusion, consequently enhancing the model’s overall accuracy in detecting objects.

### 4.3. Nbv Estimation

#### 4.3.1. 3D Gaussian Obstacle Estimation

**Figure 3:**
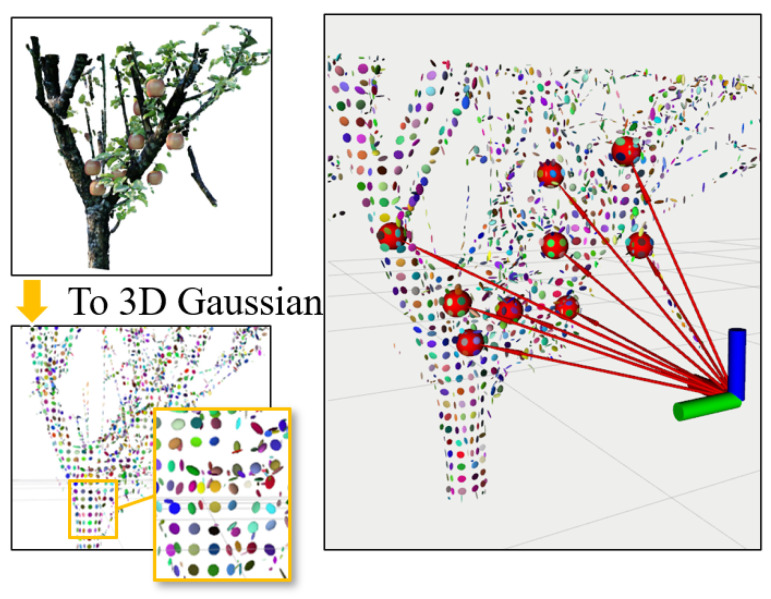
Visualization of rays from the camera to target fruits. Red spheres are the estimated fruit positions, and the red–green–blue axes represent the camera position. The red lines are the rays that are emitted by the camera to target fruits. Each colored ellipsoid represents an anisotropic Gaussian used to approximate collided objects within the scene.

We utilize anisotropic 3D Gaussians to represent obstacles around fruits. This gives us a continuously differentiable function that outputs occlusion level at any arbitrary position. As per the definition, in 3D Gaussian splatting [17], the 3D Gaussians are expressed as:

(5) $$N\left(x\right)=e^{-\frac{1}{2}{\left(X-\mu \right)}^{T}\sum^{-1}\left(X-\mu \right)}$$

where X is a given 3D position (x, y, z), μ is the mean vector, and Σ is the covariance matrix. Unlike the probability density function (PDF) of Gaussian distribution, the term $\frac{1}{{\left(2\pi \right)}^{3/2}{\left|\Sigma \right|}^{1/2}}$ is omitted here so that the center of any 3D Gaussian is always 1. See Figure 3.

In our approach, we use anisotropic Gaussians to represent ellipsoids. Within a given voxelized space, we initially filter out voxels containing point clouds. Among these voxels, we fit an ellipsoid to approximate the shape of the point cloud within each voxel. This allows us to represent the overall space using multiple anisotropic Gaussians. The mean vector of each anisotropic Gaussian represents the positions of the ellipsoid within the corresponding voxel, while the covariance matrix Σ represents the size and orientation of the ellipsoid [17].

(6) $$\Sigma =RSS^{T}R^{T}$$

Here, *R* is the orientation matrix, and *S* is the size matrix. This allows us to construct a function to express the collision index of the presence of obstacles *i* near an arbitrary point $X={\left[x,y,z\right]}^{T}$ in space.

(7) $$i\left(X\right)=\frac{1}{n}\sum \left(e^{-\frac{1}{2}{\left(X-\mu \right)}^{T}\Sigma^{-1}\left(X-\mu \right)}\right)$$

where *n* is the total number of Gaussians in the scene.

#### 4.3.2. Occlusion Level of Single Target Fruit

**Figure 4:**
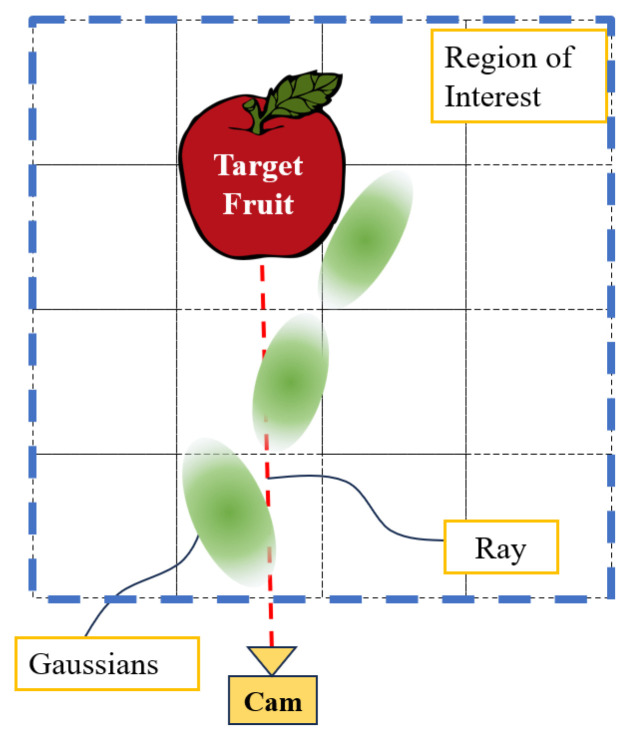
Occlusion level by anisotropic Gaussian field. The occlusion level of the given view is the integral of the collision index along the ray from the camera to the target fruit.

A ray is defined from the camera to the center of the target fruit. The collision index *i* along the ray path can be calculated, and the summation of *i* reflects the occlusion level along the ray, shown in Figure 4. This is used to represent the overall occlusion level *I* of the target fruit in the given view.

(8) $$I\left(v\right)=\int_{\mathrm{ray}\;\mathrm{path}\;v}i\left(X\right)\;dX$$

#### 4.3.3. Optimization

The goal is to select the next observation position in a manner that keeps the observation path as far away from obstacles as possible, improving the precision of estimating fruit positions and sizes.

For each fruit $f_{i}\in F_{vis}$, we first calculate the overall occlusion level *I* accordingly. The information gain of a candidate view *V* is:

(9) $$IG\left(V\right)=\sum_{\forall f\in f_{vis}}\frac{1}{I(v)}$$

The algorithm computes *I* for candidates’ viewpoints of the currently visible fruits $f_{vis}$, selecting the viewpoint that maximizes the total sum of IG values as the next optimal observation position $V^{*}$. This approach aims to optimize the observation path, keeping it as far away from obstacles as possible to improve the accuracy of estimating fruit properties. Given a set of candidate views *V*, the best next view $V^{*}$ is the one which maximizes overall information gain:

(10) $$V^{*}=\underset{V}{\mathrm{argmax}}IG\left(V\right)$$

which is optimized via gradient descent [38]. For each new view, the detection predictions are filtered using 3D non-maximum suppression (3D-NMS). This process involves selecting the most confident bounding boxes while removing redundant detections based on their spatial overlap in 3D space. Our NBV estimation is summarized in Algorithm 1. The occlusion threshold and iteration threshold are determined by experimentation.
**Algorithm 1** NBV     **Input:** Last scan’s Network outputs $F_{vis}$.
     Last scan’s camera pose ${\;}^{cam}T$.
     Under optimized fruits set $F_{u}$ from previous scans.
     **Output:** Optimized fruit set $F_{o}$ and NBV *V*. 
1:Transform Network outputs to global using ${\;}^{cam}T$.2:Update global fruits set by 3D NMS.3:Update global collision Gaussian set.4:optimization iteration n=0.5:**while** *n* < iteration threshold **do**6:    Total occlusion loss L=0.7:    $F_{u}$ extend $F_{vis}$.8:    **for** $f_{i}\in F_{u}$ **do**9:        Compute Occlusion level *I*.10:        **if** I< occlusion threshold **then**11:           $F_{u}$ pop $f_{i}$.12:        **end if**13:    **end for**14:    Compute information gain IG for $F_{u}$.15:    Compute gradient for NBV.16:    Compute NBV.17:**end while**


## 5. Implementation Details

### 5.1. Data Acquisition

**Figure 5:**
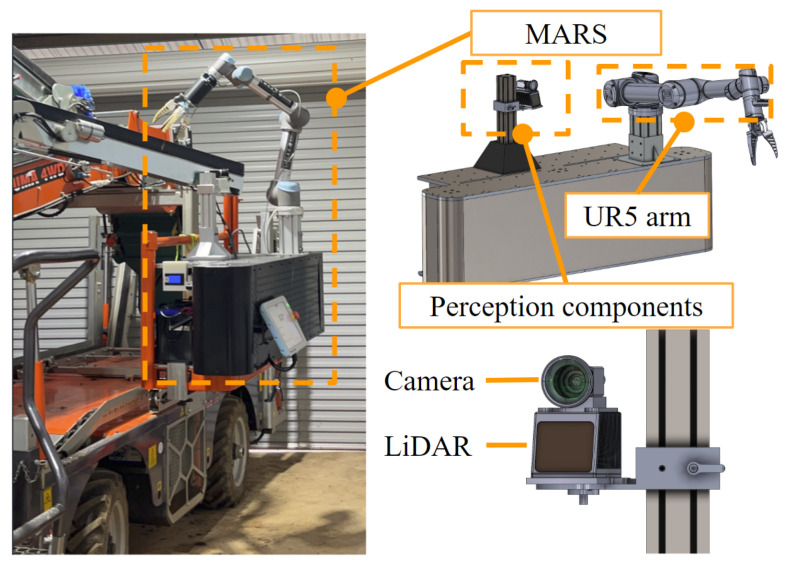
The Monash Apple Retrieve System (MARS).

Data collection was conducted using the Lidar and camera on the Monash Apple Retrieve System (MARS); see Figure 5. A dataset of 141 apple trees was acquired. The dataset contains apples with different occlusion levels, lighting conditions, and tree structures. This diversity in the dataset allows our 3D apple detection model to learn and generalize effectively in real-world scenarios. In the point cloud data collected by the original Lidar, each point only contains four values—x, y, z, and intensity—and does not contain color information. Therefore, we need to project the points of the Lidar onto the pictures collected by the camera for coloring. By precisely calibrating the camera and Lidar, we can obtain the intrinsic matrix *K* of the camera and the transformation matrix ${\;}_{L}^{C}T$ between the Lidar and camera coordinate system [22].

We can transform each point from a Lidar scan ${\;}^{L}P\left(x_{L},y_{L},z_{L}\right)$ into the camera frame to obtain the ${\;}^{C}P\left(x_{C},y_{C},z_{C}\right)$ using the following equation:

(11) $${\;}^{C}P{=}_{L}^{C}T{\;}^{L}P$$

We obtain the point in the converted camera coordinate system and then multiply the point by the camera intrinsic matrix *K* to project the point onto the image plane so that its coordinates (u,v) on the pixel plane system can be obtained. Through (u,v), we can obtain the color value of the point.

(12) $$z_{C}\left[\begin{array}{c} u\\ v\\ 1 \end{array}\right]=\left[\begin{array}{ccc} f_{x} & 0 & u_{0}\\ 0 & f_{y} & v_{0}\\ 0 & 0 & 1 \end{array}\right]\left[\begin{array}{c} x_{C}\\ y_{C}\\ z_{C} \end{array}\right]=K\left[\begin{array}{c} x_{C}\\ y_{C}\\ z_{C} \end{array}\right]$$

Here, we use the pin-hole camera model; the parameters $f_{x},f_{y}$ and $\left(u_{0},v_{0}\right)$ are the components of the camera intrinsic matrix *K* [39].

### 5.2. Data Labeling

Annotating the 3D bounding box of each apple can be a challenging task due to the arbitrary spatial positions of the apples. We utilized an open-source 3D point cloud processing software called CloudCompare [40] to address this challenge effectively. The ground truth of each apple is represented by eight corner points and one center point. To ensure compatibility and ease of training, we subsequently converted these annotated labels into the SUN-RGBD [41] dataset format. This format conversion enables us to leverage existing tools and frameworks that support the SUN-RGBD dataset, facilitating the training and evaluation processes.

### 5.3. Dataset Structure

The dataset contains three kinds of fruits, namely apple, orange, and lychee. Apple has the most data among them. Thus, in the following section, we mainly analyze the performance of apple detection. A total of 141 labeled scans of apple trees were obtained with 2283 apples and were divided into 70% for the training set, 20% for the validation set, and 10% for the test set. To avoid overfitting the detection model, sufficient data are needed, so we further utilized the open source apple point cloud dataset Fuji-SfM [6]. The dataset consists of 1455 apples, and the point cloud is generated by using structure from motion (SfM). This dataset is mixed randomly with our dataset for training only.

### 5.4. Data Augmentation

To address the problem of overfitting resulting from a limited dataset, additional steps to apply data augmentation techniques are needed [42]. Here, we randomly rotate, translate, and scale to the training set. By randomly rotating and translating the training samples in the x, y, and z axes, we introduce variations in their orientations and positions. This improves the model’s robustness and reduces the chance of overfitting.

### 5.5. IG Calculation

To achieve real-time performance, the steps for calculating IG need to be optimized. Due to the low efficiency of loops in Python, the calculation time could easily exceed 1 s if we calculate IG by accumulating collision index *i* in a for loop. Thus, we take advantage of the efficient array computation offered by the Numpy library. The dimension of (X−μ) in Equation (7) is pre-defined as $n_{f}\times n_{i}\times n_{g}\times 3$, where $n_{f}$ is the number of visible fruit, $n_{i}$ is the number of points sampled along each ray, and $n_{g}$ is the number of anisotropic Gaussians.

## 6. Results

### 6.1. Experiment Setup

The performance of different 3D detection networks in single-view detection scenarios is evaluated on our test set with metrics of average precision (AP) and average recall (AR). This is followed by a comparison of the conventional 2D detector-based fruit detection method and FF3D. To better evaluate the multi-view detection method proposed by us, we utilized a simulated environment to generate sensor data and accurate ground truth locations. This allows us to showcase the Euclidean distance differences between the detection results and the ground truth.

### 6.2. Evaluation Metrics

To evaluate the performance of 3D detection networks in a single view, AP and AR are used. We follow the evaluation standard used in [16,33,34]. The AP is calculated based on different 3D IoU thresholds.

**Figure 6:**
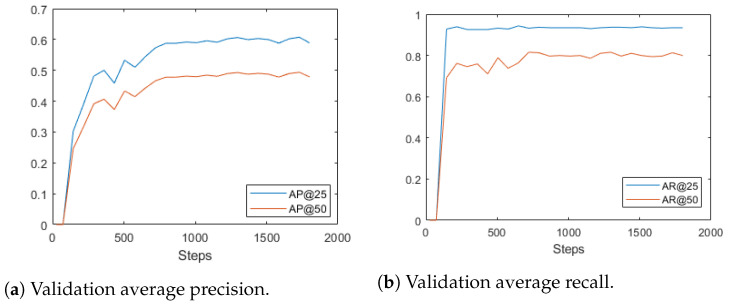
Validation results.

**Figure 7:**
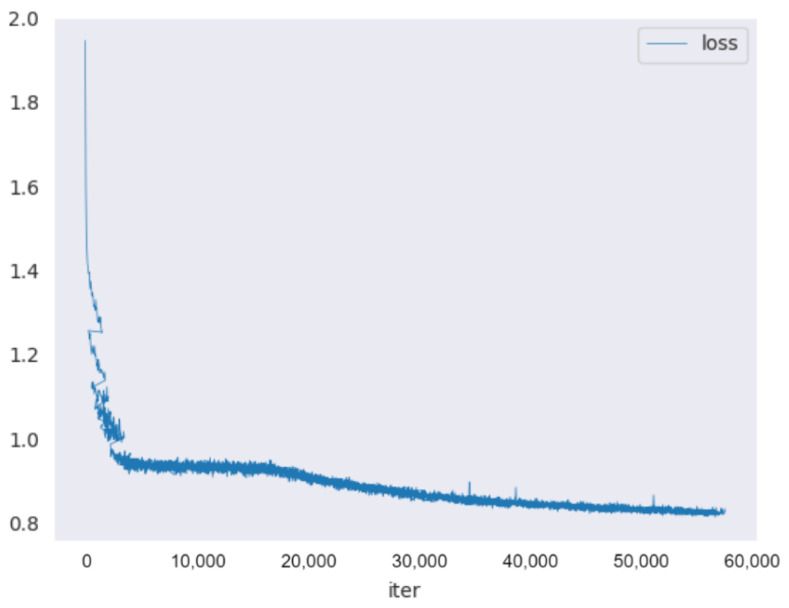
Training loss curve.

Due to the fact that most of the current fruit detection methods are 2D detection- and/or segmentation-based, we also present the comparison with the 2D detection-based method on the test set (see Table 1). The 2D method we used is [22], which utilizes 2D detection and segmentation mask to identify the corresponding depth pixel. The corresponding depth pixels are subsequently used to estimate the 3D position of apples. Along with the other four 3D detection networks, all of the methods are evaluated on our test set. The test set consists of 14 different apple trees in an orchard. The results of single-view detection are shown in Table 1; the result is averaged for 15 trials. The visualization of training loss and validation metrics are shown in Figure 6 and Figure 7, respectively. Our model was trained for 200 epochs with a learning rate of 2.000 × 10${\;}^{-5}$.

To evaluate our proposed NBV method (multi-view), we constructed a simulated apple orchard environment in the NVIDIA Omniverse Isaac Sim (version 2023.1.0) [43] NVIDIA, Santa Clara, CA, USA). This allowed us to directly access the true location and size of each fruit, which is difficult to achieve in a lab environment. To ensure a high degree of fidelity between the simulated environment and the actual apple orchard, we used neural radiation fields (NERF) [44] to obtain a model of the tree from a series of photographs of the same tree from different viewpoints. The entire simulation scenario consisted of the tree generated by NERF, high-fidelity apple models, a UR5 robotic arm (Universal Robots, Odense, Denmark), and a RealSense camera (Intel, Santa Clara, CA, USA). We conducted six tests, each with nine randomly placed apples. Each trial was limited to a total of three views for information gathering.

The metrics for multi-view are position error and size error. The position error is defined as the Euclidean distance between the estimated fruit position and the ground truth fruit position and is given in mm. The size error is defined as the difference between the estimated fruit diameter and ground truth fruit diameter in mm. The results of multi-view detection are shown in Table 2.

## 7. Discussion

First, our method achieved the best single-view performance in all four metrics, as indicated by the results in Table 1. The accuracy metric of FF3D improved by 4.5%, and the recall metric of FF3D increased by 2.7%. Importantly, our method noticeably outperformed the 2D-based baseline [22] in all metrics. This confirms that the fusion of RGB and depth information can significantly improve the object 3D detection performance under high occlusion. Compared to FCAF3D, we include an occlusion loss term that quantifies the occlusion ratio of the target fruit. This additional loss term helps the model learn more robust features that are less sensitive to occlusions. By penalizing high occlusion scenarios, the model becomes better at detecting partially visible fruits, leading to higher recall and precision. The occlusion loss forces the model to focus on more discriminative parts of the fruit that are likely to be visible even under occlusion, improving the quality of features learned by the model and making it more effective at identifying the target fruit accurately from a single view. Our model, which is deployed on the NVIDIA Jetson AGX Orin, has achieved an average inference speed of 140 milliseconds (without NBV), ensuring real-time performance.

**Figure 8:**
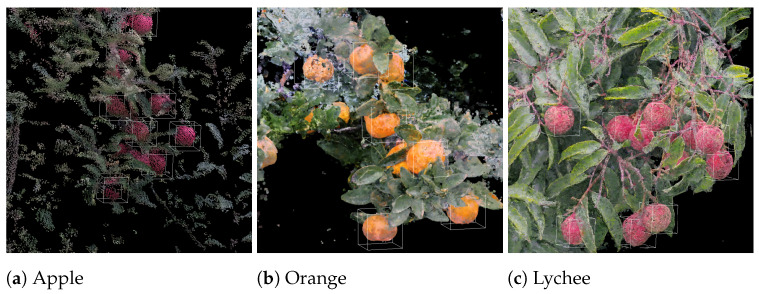
Detection results for apple, orange, and lychee.

**Figure 9:**
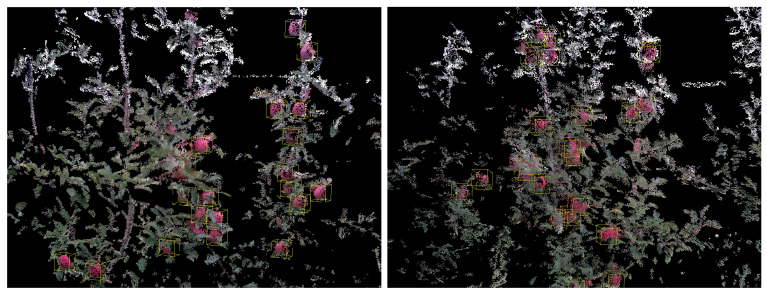
Example of the dataset; the ground truth bounding boxes of apples are marked in yellow.

To visually compare the performance of the 2D-based fruit detection method and our 3D detection-based method, we showcase two typical scenarios: moderate occlusion, and heavy occlusion, respectively. As for visualization, all of the detected apples and the colorized point cloud are visualized by ROS Rviz [45], and detected apples are represented by green spheres. The diameter of each green sphere object represents the estimated fruit diameter. See Figure 8 and Figure 9.

**Figure 10:**
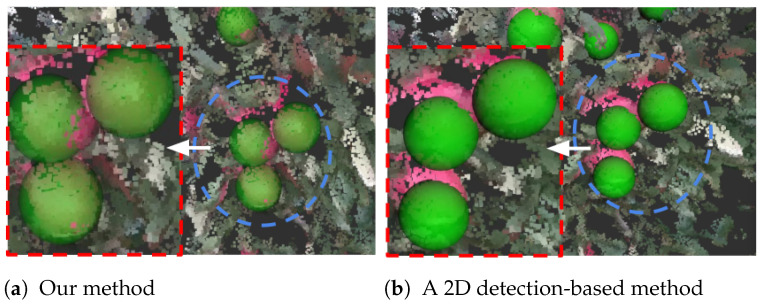
Case: moderate occlusion.

In the case of moderate occlusion, referring to Figure 10b, the difference in center estimation accuracy between the two methods is noticeable. The accuracy of fruit depth estimation experiences a noticeable decline when relying on the conventional method. As our method comprehensively utilizes spatial information, this allows it to accurately estimate the fruit centers. The method showcases a robustness that surpasses the limitations posed by occluding elements. As a result, it not only enhances the overall accuracy of fruit center estimation but also showcases its potential to address challenges associated with occlusion.

**Figure 11:**
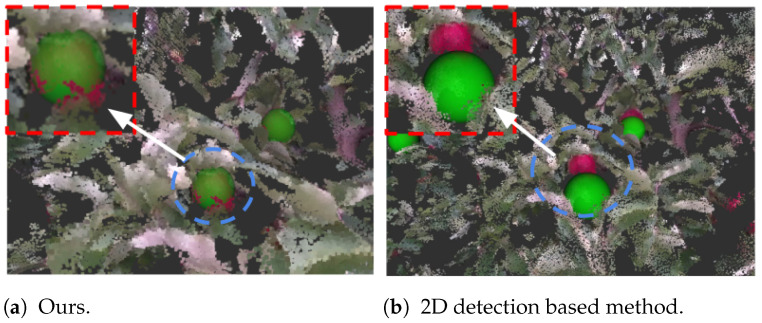
Case: Heavy Occlusion.

**Figure 12:**
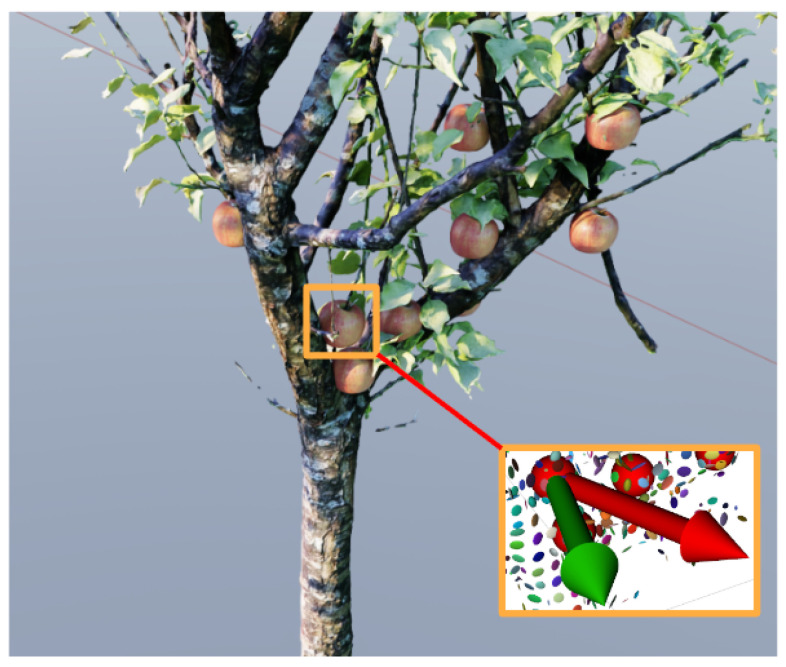
Best view of individual fruit; the red arrow is the initial view, and the green arrow is the optimized best view.

In the case of heavy occlusion, referring to Figure 11a, the difference between the two methods is substantial. A noteworthy and challenging situation encountered in real-world scenarios is when a significant distance exists between a foreground object and the target fruit. In the majority of 2D detection- and segmentation-based methods, even though they often achieve high accuracy in instance segmentation within 2D images, a minor inaccuracy in the generated mask can lead to significant errors in the estimation of fruit depth. As we can observe from Figure 11b, the estimated fruit position using the conventional method deviates noticeably from the ground truth. This depth error, in turn, has the potential to lead to failures in grasping attempts. Conversely, our method shows good accuracy under such challenging scenarios. As can be seen in Figure 11a, the green sphere that represents the estimated fruit is still fused with the surface points of the target apple seamlessly. See also Figure 12.

Second, by analyzing the position error and size error depicted in Table 2, our method exhibited significant performance improvement through NBV multi-view detection. This reaffirms that our anisotropic Gaussian-based obstacle representation is well-suited for environments with high occlusion. It is noteworthy that in complex simulated environments (obstacles represented by a total of approximately 1500 Gaussians), we achieved optimization times of less than 100 ms. This indicates that our algorithm can achieve real-time performance while retaining the majority of scene details.

Despite our method’s promising results and capabilities, it is crucial to acknowledge certain limitations that may impact its application. First, a 3D detection network requires much higher computational power, which may require a dedicated GPU for offline inference. Second, the collection and annotation of 3D datasets demand a significant amount of human resources. Finally, we observed consistent errors in both position and size estimation. This is likely attributed to errors during the annotation process that were introduced manually.

## 8. Conclusions

This research introduces the first 3D end-to-end fruit detector built on a foundation of 3D convolutional networks. A novel anisotropic Gaussian-based NBV estimator is proposed. The results demonstrate superior fruit localization accuracy and recall rate when compared with state-of-the-art 3D fruit detection methods. According to the test results, our model achieved an average precision of 76.3% and an average recall of 92.3% at an IoU threshold of 25% in single-view detection. Moreover, multi-view detection attained an average center position error of less than 3 mm in the simulation. These findings underscore the effectiveness and precision of our proposed 3D fruit detection approach. Future development will focus on enhancing NBV estimation and incorporating grasping pose estimation. Additionally, automatic generation and annotation of training data in simulated environments contribute to reducing errors introduced during manual annotation processes.

## Figures and Tables

**Table 1:** Performance of models, single view.

Method	AP@25%	AP@50%	AR@25%	AR@50%	Time (ms)
ImVoteNet	0.499	0.238	0.781	0.568	28.076
GroupFree3D	0.540	0.288	0.832	0.597	31.428
TR3D	0.591	0.302	0.838	0.586	13.647
FCAF3D	0.743	0.557	0.917	0.762	13.269
FF3D (ours)	0.763	0.602	0.923	0.789	14.871
H, Kang. et al. [22]	0.572	0.379	0.817	0.570	6.500

**Table 2:** Average fruit center position error and average fruit diameter error in mm.

Trial	Mean Position Error (mm)	Mean Diameter Error (mm)
1	5.3690	1.6218
2	6.1716	1.7870
3	5.5280	1.9342
4	5.3637	1.6682
5	5.1914	1.8015
6	5.7238	1.7097

## Data Availability

The data that support the findings of this study are available from the corresponding author upon reasonable request.
